# Divergent Secondary Metabolites and Habitat Filtering Both Contribute to Tree Species Coexistence in the Peruvian Amazon

**DOI:** 10.3389/fpls.2018.00836

**Published:** 2018-06-19

**Authors:** Jason Vleminckx, Diego Salazar, Claire Fortunel, Italo Mesones, Nállarett Dávila, John Lokvam, Krista Beckley, Christopher Baraloto, Paul V. A. Fine

**Affiliations:** ^1^Department of Integrative Biology, University of California, Berkeley, Berkeley, CA, United States; ^2^Department of Biological Sciences, Florida International University, Miami, FL, United States; ^3^Department of Ecology and Evolutionary Biology, University of California, Los Angeles, Los Angeles, CA, United States; ^4^AMAP (botAnique et Modélisation de l’Architecture des Plantes et des Végétations), IRD, CIRAD, CNRS, INRA, Université de Montpellier, Montpellier, France

**Keywords:** competitive exclusion, natural enemies, plant community assembly, resource use traits, secondary metabolites, *Protium* (Burseraceae), tropical forest diversity

## Abstract

Little is known about the mechanisms promoting or limiting the coexistence of functionally divergent species in hyperdiverse tropical tree genera. Density-dependent enemy attacks have been proposed to be a major driver for the local coexistence of chemically divergent congeneric species. At the same time, we expect local soil conditions to favor the coexistence of species sharing similar functional traits related to resource use strategies, while environmental heterogeneity would promote the diversity of these traits at both local and large spatial scales. To test how these traits mediate species coexistence, we used functional trait data for 29 species from the tree genus *Protium* (Burseraceae), collected in 19 plots (2 ha each) in the Peruvian Amazon. We characterized the presence-absence of 189 plant secondary metabolites (SM) for 27 of these species, and 14 functional traits associated with resource use strategies (RUT) for 16 species. Based on these data, we found that SM were significantly more dissimilar than null expectations for species co-occurring within plots, whereas RUT were significantly more similar. These results were consistent with the hypothesis that density-dependent enemy attacks contribute to the local coexistence of congeneric species displaying divergent chemical defenses, whereas local habitat conditions filter species with similar RUT. Using measurements of nine soil properties in each plot, we also found a significant turnover of RUT traits with increasing dissimilarity of soil texture and nutrient availabilities, providing support for the hypothesis that soil heterogeneity maintains functional diversity at larger spatial scales (from 500 m up to ca. 200 km) in *Protium* communities. Our study provides new evidence suggesting that density-dependent enemy attacks and soil heterogeneity both contribute to maintaining high species richness in diverse tropical forests.

## Introduction

Local floras in tropical rainforests often contain hundreds of tree species, including genera comprising dozens of species coexisting within the same habitats (Latham and Ricklefs, 1993; Valencia et al., 1994; Vásquez, 1997; Ribeiro et al., 1999; Wright, 2002). The coexistence of so many closely related species poses a considerable challenge to ecological theory, which predicts that, to reduce the effects of negative species interactions, each species is likely to occupy a unique niche (Hutchinson, 1957). Some researchers have speculated that closely related species are functionally similar and thus expected to share similar habitats (Harvey and Pagel, 1991). Hubbell (2005) proposed a hypothesis that consider all species as functionally equivalent (regardless of their phylogenetic relationships) and suggested that their coexistence can be explained by neutral processes and recruitment limitation rather than niche partitioning (Hubbell, 2001). Alternatively, other studies have suggested that local species coexistence is maintained by microhabitat partitioning and density-dependent processes (Janzen, 1970; Connell, 1971; Huston, 1994; Wright, 2002). For example, density-dependent attack from natural enemies (herbivores and pathogens) is a well-known mechanism that promotes coexistence at local scales (reviewed by Comita et al., 2014). If co-occurring plants share many natural enemies, they will suffer from greater density dependent attack than if they have fewer shared enemies, a process that is sometimes called “competition for enemy-free space” (Holt and Lawton, 1994). The fundamental mechanics of this process can be linked to well-known ecological end evolutionary theories like the Janzen-Connell hypothesis (Janzen, 1970; Connell, 1971) or the Resource Concentration hypothesis (Root, 1973). Among close relatives, density-dependent attacks should be more severe because the same species of insect herbivores and fungal pathogens are more likely to be shared within closely related taxa (Novotny et al., 2002; Gilbert and Webb, 2007).

Given that plant chemical defenses can mediate attacks from natural enemies, they represent important plant traits that could determine the abundance and distribution of plant species. Moreover, secondary metabolites (hereafter: SM) can represent 30–50% of a plant’s dry weight (Lokvam and Kursar, 2005), emphasizing the potential importance of selective pressures experienced by plants in response to natural enemies. Indeed, most species express dozens of metabolites that function to defend against natural enemies, and these metabolites exhibit important quantitative and qualitative variation across species (Agrawal and Weber, 2015). Because the number of shared enemies of host plants is likely to increase with the plants’ chemical similarity, it is expected that coexisting plants with shared enemies will undergo diversifying selection in chemical defenses (Ehrlich and Raven, 1964; Sedio and Ostling, 2013). As a result, at the local scale, the probability of coexistence of closely related plant species is predicted to be related to their divergence in chemical defense traits. This prediction has been supported by several studies carried out on different communities of congeneric species, for instance with the genera *Bursera* (Becerra, 2007), *Inga* (Kursar et al., 2009; Coley et al., 2018), and *Piper* (Salazar et al., 2016ab).

Contrastingly, habitat filtering can counter-balance the coexistence of functionally divergent species and instead select for similar functional traits adapted to local environmental conditions (Kraft et al., 2008; Maire et al., 2012). The traits that are generally used as proxies to investigate the effect of habitat filtering relate to plant biomechanical (e.g., wood specific gravity, bark thickness) or resource acquisition strategies (e.g., specific leaf area, leaf nitrogen content, etc., but see Violle et al., 2007). Habitat filtering can also contribute to species coexistence in highly heterogeneous environmental conditions across spatial scales. Indeed, many studies have emphasized turnover in tree species composition along environmental gradients in tropical forests (e.g., Condit et al., 2013; Baldeck et al., 2016; Vleminckx et al., 2017). Niche theory would predict that turnover in composition would be reflected by turnover in traits associated with different resource use strategies. For example, nutrient-poor white-sand forests select for species with slow growth rates, while nutrient-rich clay forests select for species with fast growth rates, suggesting that functional trait composition should correlate well with soil fertility (Fine et al., 2006). Indeed, Fortunel et al. (2014) demonstrated large-scale community-level functional turnover for 15 traits related to leaf and wood properties reflecting strategies of resource acquisition across a strong edaphic gradient in lowland Amazonian forests in both Peru and French Guiana.

Ideally, studies examining trait variation across environmental gradients would focus on monophyletic groups in which species pairs share a common evolutionary history even though they differ in key traits. Nevertheless, very few studies have investigated both SM and other functional traits within lineages to assess how different traits may contribute to local coexistence and habitat preference (Fine et al., 2013; Endara et al., 2015). An ideal study system to investigate the role of different functional traits in maintaining or limiting species coexistence is the tropical tree genus *Protium* (Burseraceae). This genus, now including the former genera *Tetragastris* and *Crepidospermum*, contains more than 150 published species, with most of them found in Amazonia (Fine et al., 2014; Daly and Fine, 2018). The alpha diversity of Amazonian *Protium* can be extraordinarily high: more than 29 species have been found in a network of 67 0.1 ha plots in the Allpahuayo-Mishana Reserve near Iquitos, Peru (Fine et al., 2005), more than 24 species exist in 25 ha of Amazonian Ecuador (Valencia et al., 2004), and 35 species co-occur in 50 ha north of Manaus, Brazil (Rankin-de-Morona et al., 1992). Moreover, as a member of the frankincense and myrrh family, *Protium* is well known for its chemical defenses, including substantial investment in both terpenes and phenolics (Fine et al., 2013; Salazar et al., 2018).

Here we combine unique datasets on SM (chemical defense traits) and resource use traits (hereafter: RUT) for 29 *Protium* species, together with detailed measures of soil properties, across a network of 19 plots located on contrasting edaphic habitats in the Peruvian Amazon. We integrate these datasets to test the following hypotheses, considering two different spatial scales:

*Hypothesis 1:* If density-dependent attack by natural enemies promotes coexistence of species that have divergent chemical defenses, we should observe a higher dissimilarity of SM than expected by chance among co-occurring *Protium* species.
*Hypothesis 2*: Given that local soil conditions are likely to select for *Protium* species with a limited range of values for RUT, we should observe a lower dissimilarity than expected by chance for these traits within plots. Among plots, we predict that turnover in soil properties would be correlated with turnover in RUT.

## Materials and Methods

### Sampling Locations and Species Richness

Between 2008 and 2010, we established 38 permanent plots in the Peruvian Amazon in three river basins: the Nanay, the Morona, and the Ucayali/Tapiche/Blanco (see Supplementary Figure S1, and Baraloto et al., 2011 for more details). These areas have similar climatic conditions and are covered by lowland tropical forest (<500 m a.m.s.l.). Within each area, we selected sites within which three broad habitat classes were found: white-sand forests, seasonally flooded forests, and terra firme forests on clay-rich soils. At each site we established two to six sample plots within different forest stands corresponding to each habitat, with at least 500 m between any two plots. All stands correspond to lowland mature forest with natural gap phase dynamics. Our plot sample method has been called “modified Gentry plots” (Phillips et al., 2003; Baraloto et al., 2013), with each plot corresponding to ten 10 m × 50 m transects that were established within a 2 ha area (see Figure 2 in Rockwell et al., 2014). All stems greater than 2.5 cm at 1.3 m height were inventoried in these transects. We also conducted additional sampling of *Protium* species across the entire 2 ha area of these plots (within or outside of the transects) to obtain one to three stems for each species observed in this genus. Voucher specimens for each collected species have been deposited in the UC herbarium (University of California, Berkeley). This protocol allowed us to be confident of the total number of co-occurring species of *Protium* within each 2 ha area; however, we did not conduct precise exhaustive counts of the number of individuals for each species in each plot, so these data are only appropriate for analyses using species presence–absence.

We tallied a total of 37 species of the genus *Protium* (Burseraceae) in the 38 plots. However, there were only 19 plots, located on white-sand (*n* = 9) and clay terra firme (*n* = 10), in which at least two species were present and for which data on both SM and RUT were available (see next sections). Supplementary Table S1 presents the list of all the *Protium* species (29 in total) present in these 19 plots, indicating species for which SM and/or RUT data were available. Six species were present in at least 10 plots (maximum 13 plots): *Protium apiculatum, P. calanense, P. calendulinum, P. crassipetalum, P. opacum*, and *P. paniculatum*. Four species were present in two plots only: *Crepidospermum goudotianum, P. grandifolium, P.* sp. nov. *G5* (Salazar et al., 2018) and *P. urophylliudium* (see Supplementary Table S1 for details).

### Soil Analyses

Soils were characterized in white-sand and clay terra firme plots. We analyzed the soil texture (percentages of sand, silt, and clay), the amount of three bioavailable cations (Ca, Mg, and K), C/N ratio, the available phosphorus and the nitrate content. The variables were measured using ten bulked 0–15 cm depth soil cores, one collected in each of the ten transects of each plot. The ten soil cores were combined into a single 500 g sample that was dried at 25°C to constant mass, and sieved to 2 mm. Samples were shipped within 3 months for physical and chemical analyses at the University of California, Davis DANR laboratory (for full details on laboratory protocols, see Baraloto et al., 2011). The edaphic heterogeneity among plots was decomposed using a principal component analysis (hereafter, “PCA_soil_”), after standardizing and normalizing all variables. Prior to the analysis, we removed variables that were overly collinear with others, based on their variance inflation factor (VIF, Lin et al., 2011). Variables having a VIF > 10 were eliminated (Kutner et al., 2004). The latter procedure generated a soil dataset composed of six remaining variables (P, NO_3_^–^, C/N, K, Ca and the percentage of silt). The PCA_soil_ revealed a single axis (explaining 43.1% of the overall soil inertia among plots) for which the observed eigenvalue was higher than the one obtained from a broken stick distribution (Cangelosi and Goriely, 2007). Axis 1 scores were significantly correlated to all soil variables except the percentage of silt (Supplementary Table S2). The biplot presenting the projection of the plot scores on axes 1–2 of the PCA_soil_ is shown in Supplementary Figure S2.

### Secondary Metabolites

To test how *Protium* community assembly was related to the SM of *Protium* species, we used a new dataset representing an exhaustive characterization of SM for each *Protium* species, using GC/MS for volatile, low-molecular weight compounds and HPLC-MS/ELSD for non-volatile, high-molecular weight compounds (see Appendix S1 and Salazar et al., 2018). Each species was sampled using leaves from six to ten juvenile individuals in the Allpahuayo-Mishana Reserve near Iquitos, Peru (the same Reserve as for two of the plots in this study). For 14 of the species, we also sampled the chemistry of six to ten juvenile individuals from the Ducke Reserve and the Campinas Reserves near Manaus, Brazil (2500 km distant). We found very small intraspecific variation in leaf chemistry in Iquitos and Manaus. Although the relative abundance of some SM changed slightly across sites, all species maintained extremely high qualitative consistency in their chemical composition within species, a pattern also found for other tropical tree genera in a recent study by Endara et al. (2018). Even when 2500 km distant, populations of the same species shared over 95% of SM (Supplementary Figure S3). Nevertheless, it is important to note that our secondary metabolite data for this paper were collected from juvenile *Protium* trees located in a single forest (the Allpahuayo-Mishana National Reserve near Iquitos Peru), and we assume that the shared chemicals that we found in individuals from Allpahuayo-Mishana apply to all individuals of those species across our plot network.

Previous studies have already emphasized high consistency of leaf chemical composition among conspecific plants in the species-rich genera *Eugenia, Inga, Ocotea* and *Psychotria* (Bixenmann et al., 2016; Wiggins et al., 2016; Sedio et al., 2017). In addition, at the community-scale, Asner et al. (2014) found high ratios of inter- to intraspecific variation for leaf chemical traits assessed from spectral data. And within the genus *Inga*, Endara et al. (2018) demonstrated the accuracy of chemical profile characterization (“chemocoding”) as a tool to identify different species. All of these studies suggest that intraspecific variation in chemical composition remains much lower than interspecific variation. Thus we believe that our approach to compare community surveys using presence-absence of secondary metabolites for each species can be considered appropriate to compare chemical community composition and community similarity, especially given the costly alternative to characterize the defense chemistry of each individual plant sample (Asner et al., 2014).

In addition, for each species, we sampled both young and mature leaves including those that experienced little or no prior herbivore attack as well as those that exhibited evidence of herbivore damage. Thus, our chemical characterization dataset likely includes both constitutive and induced defenses for each species, although it is impossible from our methodology to identify which chemicals are induced and which ones are constitutive.

It is also important to note that our secondary metabolite data were collected from juvenile plants (1–2 m tall) only, while our co-occurrence data includes adult trees in addition to juveniles. We have only limited data on secondary metabolite investment from adult *Protium* trees, yet for two of the species in our dataset we found very similar chemical composition in juveniles compared with adults (Lokvam et al., 2015). Moreover, a recent analysis found highly significant positive correlations for phenolic investment between adults and juveniles for 23 species of *Protium*, suggesting that ontogenetic changes in this important class of secondary metabolites are minor in this genus (Supplementary Figure S4). Nevertheless, further studies are needed to test how chemical composition changes from the juvenile to the adult stage among all *Protium* species.

In order to relate community assembly to secondary chemical composition of *Protium* species, we therefore are making a large assumption that *Protium* chemistry does not substantially vary across the plots in our study nor with ontogeny. It is important to note that the dataset that we generated of six to ten juvenile individuals per species within one forest took over 3 years of work; thus it would be a major undertaking to conduct a complete chemical characterization of all of the individuals from all of the plots included in this study. We acknowledge that geographic, environmental, and ontogenetic variation among plots could indeed influence the secondary metabolite composition of the *Protium* species within the plots; however, we believe that it is valuable to test the species-level secondary metabolite composition with patterns of community assembly because the hypothesis of divergent secondary metabolites is compelling and such detailed secondary metabolite datasets for so many species within a tropical tree genus are so rare. Further details on the chemical extraction protocols are available in Appendix S1.

In order to test the hypothesis that divergent chemical defenses are selected among co-occurring species (hypothesis 1), we used the presence-absence of 189 secondary metabolites (SM) that have been previously characterized for 31 *Protium* species (see Salazar et al., 2018 and Appendix S1). The great majority of SM belonged to two main families of secondary compounds, terpenoids (*n* = 89) and phenolics (*n* = 97). 27 out of the 29 species present in the 19 plots had available SM data. These 27 species had, on average, 31.4 different secondary metabolites (±*SD* = 12.8). Each metabolite was present in at least two species (Salazar et al., 2018). Because the great majority of herbivores feed on multiple species, we argue that shared chemicals (present in at least two species) are more relevant to testing how chemical divergence is related to coexistence at the local scale. Unique chemicals were also characterized by our methodology but are by definition only present in one species, and thus including them in our analyses would only introduce noise (even though they potentially could have anti-enemy function). We submit that shared chemicals represent a common currency for species that share natural enemies (Agrawal and Weber, 2015) and provide a more conservative estimate of species divergence than if we included all chemicals. Therefore, we compared only the presence-absence of shared SM among coexisting species to test if plants were more chemically divergent than random expectations (see next sections). Salazar et al. (2018) found that the great majority of insect herbivores that attacked *Protium* in Peru fed on more than one species of *Protium* host plant, with 11 as the average number of host plant species per insect herbivore. We do not present a PCA for the 189 chemicals since their number was so much greater than the total number of species (*n* = 29) (Legendre and Legendre, 2012), and because the two first PC axes did not represent more than 20% of the SM inertia (not shown).

### Functional Traits Related to Resource Acquisition and Mechanical Resistance

In order to test hypothesis 2, we used data on 14 resource use traits (RUT) that have already been characterized for 16 *Protium* species (see Table 1 in Fortunel et al., 2012). Three traits relate primarily to plant mechanical resistance: bark thickness, stem and root wood density. The other traits relate to both resource capture strategies and structural defense against natural enemies. They comprise leaf chlorophyll content, leaf thickness, leaf toughness, leaf tissue density, specific leaf area (SLA), leaf area (LA), leaf contents in carbon, nitrogen, phosphorus and potassium, and leaf δ^13^C composition. δ^13^C in particular is used as a proxy for water use efficiency (Farquhar et al., 1989; Dawson et al., 2002). The protocol used to measure all the RUT is detailed in Fortunel et al. (2012). In order to make intraspecific measures comparable among traits, each trait was standardized (*z*-scores, Zar, 1999). They were then normalized, and a principal component analysis on the traits’ correlation matrix was performed in order to decompose the overall RUT inertia into few axes representing composite trait variation (“PCA_RUT_”). As we did for soil variables, we calculated a variance inflation factor prior to the PCA_RUT_. However, this procedure only allowed two traits to be removed and did not reduce the number of significant PC axes, while the first PC axis only explained 2% more RUT variation. We therefore retained all RUT in our analyses. Each of the first four axes (representing 26.5, 23.1, 14.1, and 10.7% of the overall RUT variation) of the PCA_RUT_ showed larger variation (eigenvalues) than expected by a broken stick model. Correlations between each trait and each of the three first PC axes are presented in Supplementary Table S3. The fourth axis was not taken into account in our tests as it was only significantly correlated with leaf carbon content (which was significantly correlated to the third axis). The PCA_RUT_ biplots representing correlations between traits and the projection of plots on axes 1–2 and 3–4 are presented in Supplementary Figure S5. There were 14 species that had both SM and RUT data available (50 and 87.5% of the species with SM and RUT data available, respectively; see Supplementary Table S1). These 14 species were present in 18 out of the 19 (95%) plots.

### Testing Within-Plot Functional Dissimilarity

To test hypothesis 1 (SM are more dissimilar than expected by chance among co-occurring species), we first calculated the mean observed dissimilarity (1- Jaccard index, Zhang et al., 2016) of SM composition (presence–absence) between co-occurring species within plots. For hypothesis 2 (RUT are more similar than expected by chance among co-occurring species), we calculated the mean distance (Euclidean) of RUT among co-occurring species within plots. This latter distance was calculated for the assemblages of traits that were significantly correlated with each of the three first axes of the PCA_RUT_ (see section “Results” for details), separately. Observed distance values were compared to a distribution of expected distance values obtained by using a null model that does not assume trait divergence or convergence. In this model, we replaced, in each plot, the *N* observed species by *N* randomly sampled ones (with replacement) from the list of all species names (each species equally represented, assuming that all of them have an even chance to be present in each plot). The SM or RUT composition of each species was kept intact, thereby assuming that both trait types and have been conserved within species. We then computed the median of the difference between the observed and expected distance values among plots (*n* = 19). The median was preferred to the mean due to the limited number of plots (*n* = 19) and to lower the effect of outlying values (but we also controlled for outliers following the method used by Zuur et al., 2010). This procedure was repeated 1000 times, and a *p*-value was then obtained as the proportion of median values higher (if testing whether within-plot SM are more divergent than expected by chance, hypothesis 1) or lower than zero (if testing whether RUT are more convergent than expected by chance, hypothesis 2).

### Testing Functional Turnover With Spatial and Soil Distance

In order to further test hypothesis 2, functional turnover (of SM and RUT) among plots was calculated for each pair of plots using the TAU_st_ statistic. The latter is exactly analogous to the Π_st_ statistic in phylogenetic turnover analyses and has been specifically designed for analyzing functional turnover using presence absence data (Hardy and Senterre, 2007). TAU_st_ is calculated as followed for each pair of plots:

$$\begin{array}{c} {\mathrm{TAU}}_{\mathrm{st}}\;=\;1\;-\;D_{w}/D_{a} \end{array}$$

Where *D* quantifies the mean functional distance between distinct species located within a same plot (*D*_w_) or between distinct species among two different plots (*D*_a_). TAU_st_ thus measures the proportion of functional diversity expressed among plots (compared to within plots). Positive TAU_st_ values indicate functional clustering (species from two different plots are more functionally distant, on average, than species from the same plot), while negative values indicate functional overdispersion (species from two different plots are functionally more similar, on average, than species from the same plot). Pairwise TAU_st_ values were plotted against spatial distance (log transformed) and soil distance (Euclidean) between plots. Soil distance was calculated using the five variables that were significantly correlated with plot scores along the first axis of the PCA_soil_: C/N, NO_3_^–^, P, K, and Ca. We then tested the correlation between TAU_st_ and spatial/soil distance using a Mantel test (999 randomizations).

All statistical analyses described in the methods were performed in R statistical environment (R Development Core Team, 2017), using packages *stats* (R Development Core Team, 2017), *vegan* (Oksanen et al., 2015), *car* (Fox and Weisberg, 2011), *usdm* (Naimi, 2013), *ape* (Paradis et al., 2004), *ade4* (Dray and Dufour, 2007), and *SpacodiR* (Eastman et al., 2013). The *Protium* species presence-absence, SM, RUT and soil data as well as the R code used to test within-plot trait dissimilarity and perform turnover analyses are available in Appendices S2–S6.

## Results

### Within-Plot Functional Dissimilarity

**FIGURE 1:**
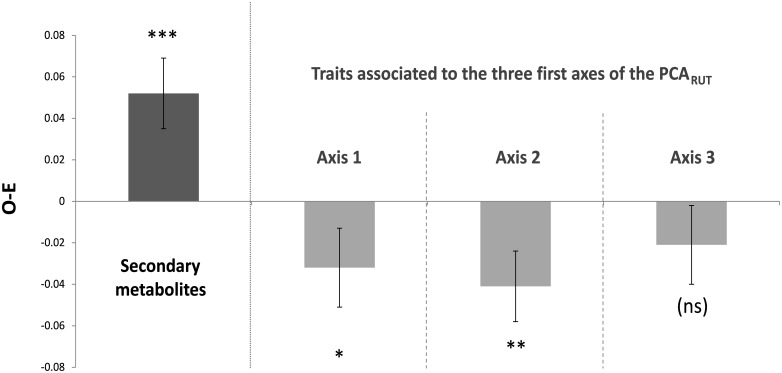
Results of the within-plot functional dissimilarity analyses. O-E: mean (±*SD*) of the 1000 values obtained by calculating the median of the difference (among the 19 plots) between the observed and expected functional dissimilarity. Asterisks indicate whether O-E was significantly positive or negative, or neither of both (ns). ^∗^*p* ≤ 0.05, ^∗∗^*p* ≤ 0.01, ^∗∗∗^*p* ≤ 0.001. “axis 1” means that Euclidean distances were computed using resource use traits that were significantly correlated with the first axis of the PCA_RUT_ (idem for axes 2 and 3).

We found significant divergence in secondary metabolites (SM) within plots, as indicated by a significantly positive median of the difference between the observed and expected SM distance values among plots (*P* < 0.001) (**Figure 1**). Conversely, significant convergence (negative median values) within plots was observed for assemblages of resource use traits (RUT) that were significantly correlated with the first and second axes of the PCA_RUT_ (*P* = 0.021 and 0.008, respectively), but not with the third axis (*P* = 0.17). The traits from the first and second axes of the PCA_RUT_ involved every RUT except bark thickness, leaf thickness and leaf carbon content (79% of the traits, see Supplementary Table S3). More particularly, leaf area, leaf phosphorus content, leaf toughness, leaf chlorophyll content, leaf δ^13^C and leaf thickness had the highest average correlations with the two first axes of the PCA_RUT_ when weighting correlations by the eigenvalues of these axes (see Supplementary Table S3).

### Functional Turnover Among Plots

**FIGURE 2:**
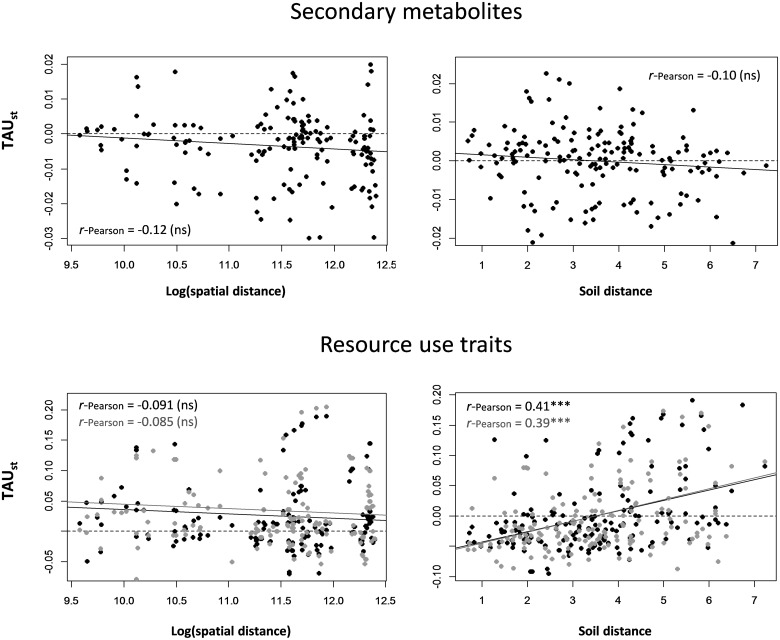
Functional turnover among all pairs of plots (TAU_st_), calculated for secondary metabolites and resource use traits, plotted against spatial distance (log-transformed) and soil distance (after removing spatial effect) between plots. For resource use traits, black and gray dots correspond to TAU_st_ values calculated using traits that were significantly correlated with axes 1 and 2 of the PCA_RUT_, respectively. Correlation values between TAU_st_ and spatial/soil distance are represented in each graph. Asterisks indicate whether the Mantel test of each correlation was significant (^∗∗∗^*P* ≤ 0.001) or not (ns). Soil distance was calculated using the five soil variables (C/N, P, NO_3_^–^, K, Ca) that were significantly correlated with the first axis of the PCA_soil_.

Significant turnover among plots was found with soil distance for RUT but not for SM. Pairwise TAU_st_ values quantifying SM turn-over were negatively but not significantly correlated with spatial and soil distance between plots (*r*-Pearson = -0.12 and -0.10, with *P* = 0.15 and 0.19, respectively, Mantel test, **Figure 2**). TAU_st_ values calculated using the assemblages of RUT that were significantly correlated with the first and second axes of the PCA_RUT_ (i.e., all traits except bark thickness, leaf thickness and leaf C content) were significantly and positively correlated with soil distance (*r*-Pearson = 0.41 and 0.39, respectively, with *P* < 0.001, **Figure 2**). They were, however, not significantly correlated with spatial distance between plots (*r*-Pearson = -0.091 and -0.085, with *P* = 0.25 and 0.22, respectively, **Figure 2**). No significant correlation of TAU_st_ values with soil or spatial distance was observed with the assemblages of RUT significantly associated with axes 3 and 4 of the PCA_RUT_ (not shown).

## Discussion

We found significant divergence in secondary metabolite (SM) composition, but convergence in resource use traits (RUT) for co-occurring *Protium* tree species at local scales. These results were consistent with our hypotheses, which posit (i) that divergent chemical defenses are selected among co-occurring *Protium* species, possibly in response to density-dependent enemy attacks; and (ii) that habitat filtering tends to promote similarity in traits related to resource use strategies. Among plots, we found a significant turnover of resource use traits with dissimilarities in soil texture and nutrient availabilities, providing support for the hypothesis that soil heterogeneity maintains functional diversity patterns in *Protium* at larger spatial scales.

### Local Divergence of Secondary Metabolites vs. Convergence of Resource Use Traits

Shared secondary metabolites were significantly more overdispersed than expected by chance among co-occurring *Protium* species (**Figure 1**). This is consistent with the idea that, at the neighborhood scale, species from the local pool which produce defense chemicals that are highly similar to the resident *Protium* community would suffer greater density-dependent attack than chemically divergent species (Becerra, 2007; Sedio and Ostling, 2013), thereby promoting species coexistence (Wright, 2002; Leigh et al., 2004). Other studies on the chemical diversity of tropical woody plants have reported similar results; for example in Coley et al. (2018) for *Inga* (Fabaceae), Sedio et al. (2017) for *Inga, Eugenia* (Myrtaceae), *Ocotea* (Lauraceae) and *Psychotria* (Rubiaceae), and in Salazar et al. (2016a,b) for *Piper* (Piperaceae). The consistency of interspecific chemical divergence among congeners in such distantly related Angiosperm families suggests that competitive exclusion mediated by natural enemies may represent a major driver of species assembly at the community level in tropical forests. Our study further confirms and expands on these results by showing, for the first time, that these processes are also found at larger geographic scales. Here, we show divergent chemical traits within *Protium* communities replicated over 19 forest plots across several hundred square kilometers.

*Protium* species were characterized by a convergence of RUT associated with the two first axes of the PCA_RUT_ (explaining 49% of the RUT variation among species) (**Figure 1**), suggesting that a limited range of plant strategies related to resource use and tissue protection (mechanical resistance) is selected to face the particular soil conditions occurring within each plot. Looking at the functional composition at the whole community level for 15 traits (including the 14 traits studied here), Fortunel et al. (2014) also found trait convergence within terra firme and white-sand soils, and in particular showed that white-sand communities displayed denser wood, tougher leaves and lower leaf nutrient contents than communities on terra firme soils. In a different community-level study involving 1100 woody plant species, Kraft et al. (2008) found that values of specific leaf area, leaf N content, leaf area and diameter were more evenly distributed than expected by chance for trees located within 20 m × 20 m clayey-soil plots in Ecuadorian rainforest.

### Soil Heterogeneity Increases the Diversity of Resource Use Traits

In agreement with our second hypothesis, we found that the turnover of RUT associated with axes 1 and 2 of the PCA_RUT_ was significantly and positively correlated with soil distance (**Figure 2**). The latter distance was computed with all soil variables except the percentage of silt (Supplementary Table S2). The two first axes of the PCA_RUT_ were significantly explained by all traits except bark thickness, leaf thickness and leaf carbon content. This provides additional evidence that soil filtering contributes to the sorting of functional traits related to hydric stress resistance (δ^13^C), nutrient capture strategies (leaf K, P, and N contents, chlorophyll content, leaf area and SLA), and to plant tissue resistance traits (bark thickness, stem and root wood densities, leaf toughness) in tropical forests (ter Steege et al., 2006; Kraft et al., 2008; Lebrija-Trejos et al., 2010; Paine et al., 2011; Katabuchi et al., 2012; Liu et al., 2012; Fortunel et al., 2014). However, whereas these studies compared entire tree communities, here we found similar patterns within a single genus (*Protium*). Our result therefore underlines how combinations of traits that are selected as optimal strategies have evolved repeatedly in white-sand and clay specialist species from many angiosperm lineages. It appears that the same processes have influenced speciation over a large window of time, which has resulted in both recently evolved and ancient associations to different soil types in the Amazon (Fine and Baraloto, 2016).

## Conclusion

Our results support the idea that the local coexistence and assembly of tropical tree species in the hyperdiverse genus *Protium* is influenced by divergent selection of secondary metabolites, which is consistent with the hypothesis of density-dependent attacks by shared natural enemies. At the same time, functional traits associated with resource use strategies and tissue resistance displayed lower variation than expected by chance, showing that soil filtering promotes local trait similarity. Finally, our results suggest that the turnover of functional traits in response to edaphic heterogeneity contributes to species coexistence at the landscape and regional scale.

## Author Contributions

JV led the data analyses and led the writing of the manuscript with the help of PF, CF, DS, and CB. JV, DS, and PF designed the statistical protocol. CF and CB collected the resource use traits. DS, KB, and JL helped to design protocols for laboratory work and collected valuable data on *Protium* species identity (barcoding) and secondary metabolites. IM, ND, PF, and CB designed field protocols and collected valuable field data on *Protium* species occurrences in the plot network.

## Conflict of Interest Statement

The authors declare that the research was conducted in the absence of any commercial or financial relationships that could be construed as a potential conflict of interest.
