# Supplementary material for: Divergent Secondary Metabolites and Habitat Filtering Both Contribute to Tree Species Coexistence in the Peruvian Amazon
Source: Front Plant Sci. 2018 Jun 19;9:836. doi: 10.3389/fpls.2018.00836 (PMC6018647; doi:10.3389/fpls.2018.00836)
Supplement: Supplementary file 1 [file Table_1.docx]

**Supplementary tables S1 to S3**

**Table S1.** List of the 29 *Protium* species (in alphabetic order) recorded in the 19 plots, indicating (crosses) species for which secondary metabolites and/or resource use traits data were available. The third column (Nr plots) indicates the number of plots where species were present.

|  | ***Secondary metabolites (SM)*** | ***Resource-use traits (RUT)*** | ***Nr plots*** |
| --- | --- | --- | --- |
| *Crepidospermum goudotianum* | X |  | 2 |
| *Protium altsonii* | X | X | 5 |
| *Protium alvarezianum* |  | X | 6 |
| *Protium amazonicum* | X |  | 8 |
| *Protium apiculatum* | X | X | 10 |
| *Protium calanense* | X | X | 10 |
| *Protium calendulinum* | X | X | 10 |
| *Protium crassipetalum* | X | X | 10 |
| *Protium decandrum* | X |  | 3 |
| *Protium divaricatum* | X |  | 9 |
| *Protium ferrugineum* | X |  | 6 |
| *Protium gallosum* | X | X | 6 |
| *Protium grandifolium* | X |  | 2 |
| *Protium hebetatum* | X | X | 7 |
| *Protium klugii* | X |  | 5 |
| *Protium krukovii* | X |  | 3 |
| *Protium laxiflorum* |  | X | 5 |
| *Protium nodulosum* | X | X | 6 |
| *Protium opacum* | X | X | 13 |
| *Protium pallidum* | X | X | 4 |
| *Protium paniculatum* | X | X | 12 |
| *Protium sagotianum* | X |  | 3 |
| *Protium spruceanum* | X |  | 8 |
| *Protium subserratum MPH1* | X | X | 9 |
| *Protium subserratum MPH2* | X |  | 7 |
| *Protium sp. nov. G5* | X |  | 2 |
| *Protium trifoliolatum* | X | X | 7 |
| *Protium urophyllidium* | X | X | 2 |
| *Tetragastris panamensis* | X |  | 4 |

**Table S2**. Correlations between the first significant axis of the PCA_soil_ (performed with six variables obtained after removing collinearities, see methods) and each of all (nine) soil variables. The significance of the correlations (permutation test, using 999 randomizations) is indicated by asterisks: *p ≤ 0.05; **p ≤ 0.01; ***p ≤ 0.001.

|  | ***PC axis 1*** |
| --- | --- |
| ***C/N*** | -0.835*** |
| ***NO_3_^-^*** | 0.642** |
| ***P*** | -0.545* |
| ***K*** | 0.615** |
| ***Ca*** | 0.853*** |
| ***Mg*** | 0.809*** |
| ***Percent sand*** | -0.822*** |
| ***Percent silt*** | 0.287 |
| ***Percent clay*** | 0.657** |

**Table S3.** Pearson correlation values between each of the three significant axes of the PCA_RUT_ and each resource-use trait (RUT). The last column indicates the average weighted correlation (AWC) between each trait and the two first axes of the PCA_RUT_ (i.e. the mean of the two correlation values obtained by multiplying correlations in columns 1 and 2 by the eigenvalues of PC axes 1 and 2, respectively). The third axis was not taken into account to calculate AWC, as O-E was not significant for traits significantly correlated to axis 3 of the PCA_RUT_ (see Fig. 1). Significance of correlation test (permutation test of the correlation coefficient, using 999 randomizations) is indicated by asterisks: **p* ≤ 0.05; ***p* ≤ 0.01; ****p* ≤ 0.001.

|  | ***PC axis 1*** | ***PC axis 2*** | ***PC axis 3*** | ***AWC*** |
| --- | --- | --- | --- | --- |
| ***Bark thickness*** | 0.099 | -0.422 | 0.686** | 6.185 |
| ***Stem wood density*** | -0.582* | 0.159 | 0.474 | 9.548 |
| ***Root wood density*** | -0.052 | 0.643** | -0.248 | 8.116 |
| ***Leaf chlorophyll content*** | -0.29 | -0.717** | 0.082 | 12.124 |
| ***Leaf thickness*** | 0.48 | -0.374 | -0.306 | 10.680 |
| ***Leaf toughness*** | 0.537* | -0.664** | -0.203 | 14.784 |
| ***Leaf tissue density*** | -0.471 | -0.545* | -0.156 | 12.536 |
| ***Specific leaf area (SLA)*** | 0.329 | 0.685** | 0.512* | 12.271 |
| ***Leaf area*** | 0.647** | -0.626** | 0.232 | 15.803 |
| ***Leaf C content*** | 0.381 | -0.038 | -0.583* | 5.487 |
| ***Leaf N content*** | 0.628** | 0.039 | 0.528* | 8.771 |
| ***Leaf P content*** | 0.599* | 0.559* | 0.08 | 14.393 |
| ***Leaf K content*** | 0.692** | 0.067 | 0.131 | 9.943 |
| ***Leaf δ^13^C*** | 0.786*** | 0.092 | -0.293 | 11.477 |
