# Supplementary material for: Divergent Secondary Metabolites and Habitat Filtering Both Contribute to Tree Species Coexistence in the Peruvian Amazon
Source: Front Plant Sci. 2018 Jun 19;9:836. doi: 10.3389/fpls.2018.00836 (PMC6018647; doi:10.3389/fpls.2018.00836)
Supplement: Supplementary file 2 [file Data_Sheet_1.docx]

**Supplementary figures S1 to S5**

**
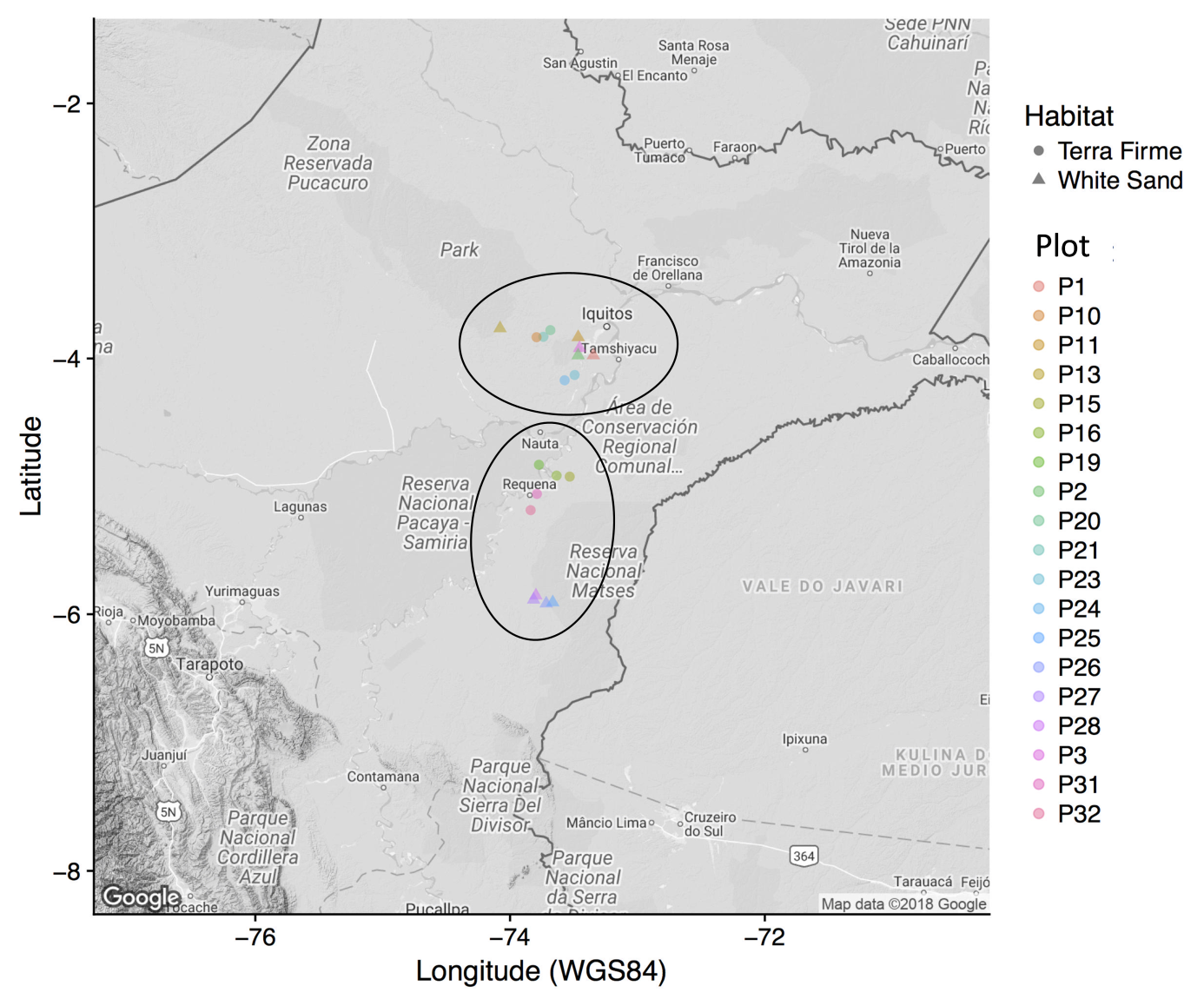
**

**Fig. S1.** Map of the 19 plots located in the Loreto Amazon region (Peru), representing the two (encircled) areas where the 19 plots used in our analyses were established: the Allpahuayo-Mishana Reserve in the north and the Jenaro Herrera Center of Investigation and the Matsés Reserve in the south. Plots represented by triangles and dots correspond, respectively, to plots located on white sands and terra firme.


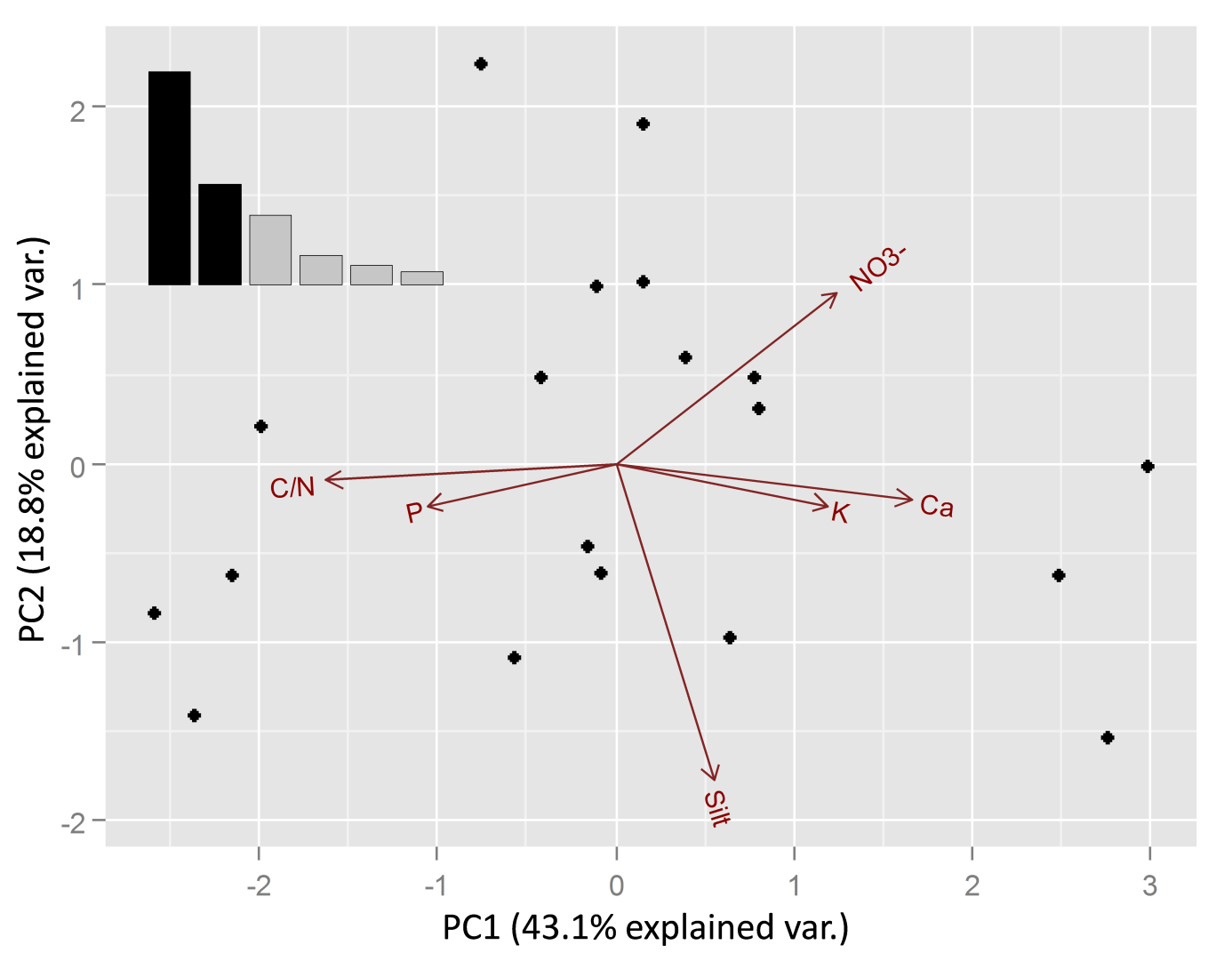


**Fig. S2**. Biplot of the principal component analyses on six soil variables (PCA_soil_), showing the projection of the 19 plot scores (dots) on PC axes 1 and 2. Black histograms represent the eigenvalues of the projected axes.


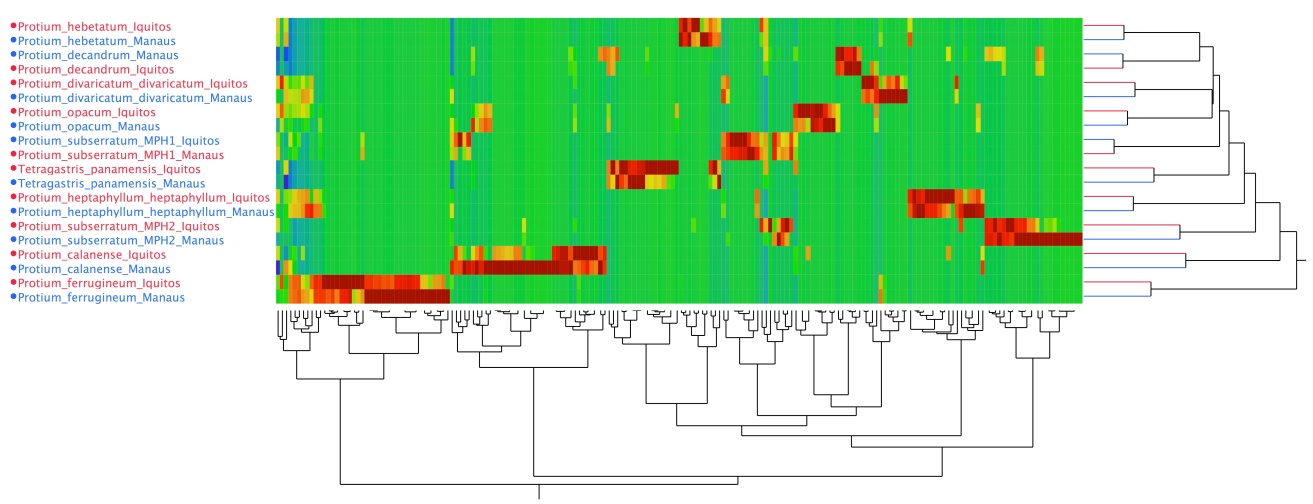


**Fig. S3.** Chemical based dendrogram and heatmap for 10 *Protium* species collected at two distant geographical locations (Iquitos, Peru and Manaus, Brazil). Pixels in the heatmap represent *Protium* secondary metabolites. The color scale represents the relative abundance of the metabolite, from blue = very low, to green = intermediate, and red = very high. Metabolite data was normalized by metabolite (rows in the heatmap). Dendrogram at the right of the figure clusters *Protium* samples by chemical similarity; samples colored in red were collected from Iquitos; samples in blue were collected in Manaus. Finally, dendrogram at the bottom of the figure shows the similarity in the metabolites as it is related to the amount on information used by the chemical clustering algorithm.

**
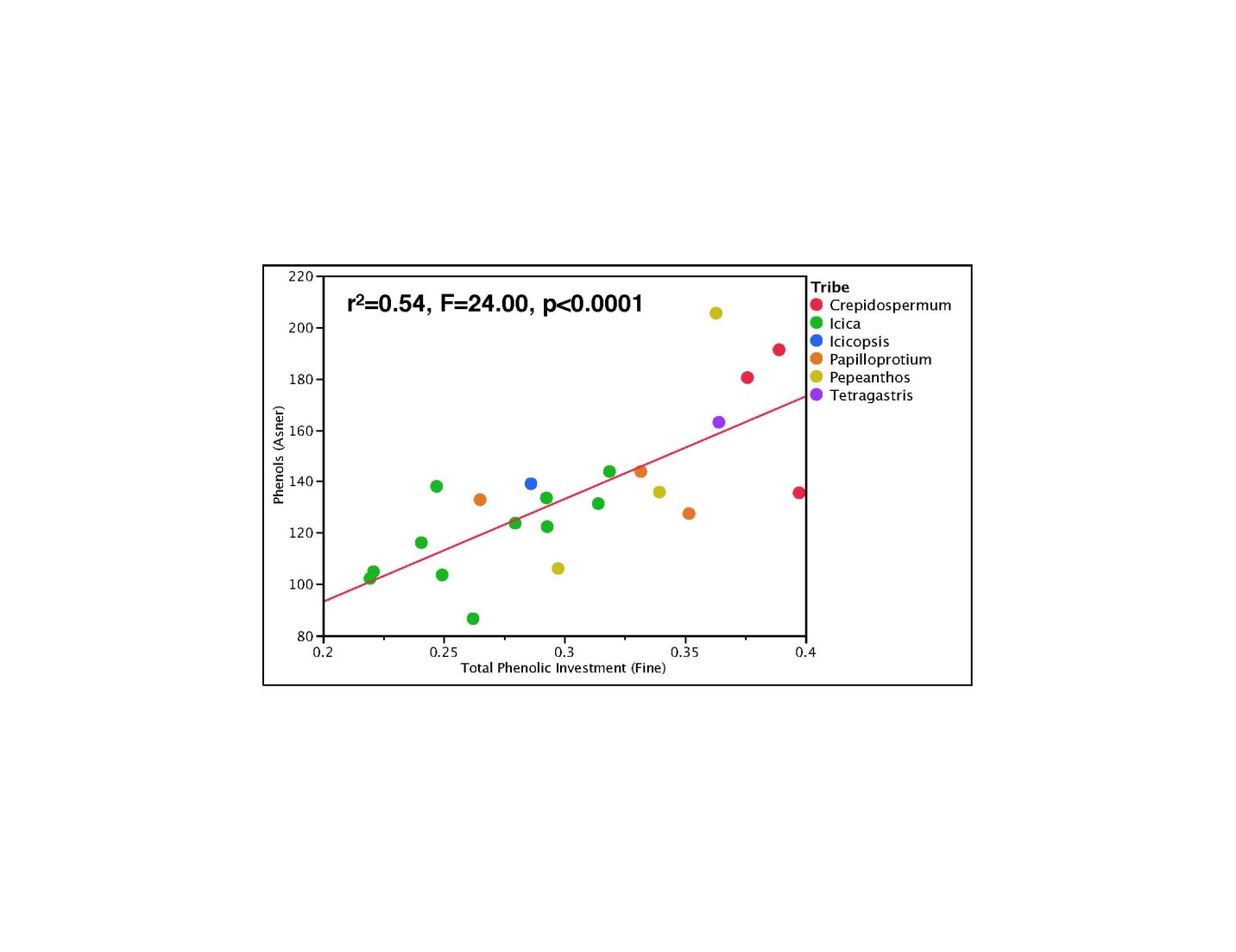
Fig. S4**. Correlation between total phenols data from canopy *Protium* trees (Asner et al. 2014) and measurement of total phenolic investment in juvenile *Protium* trees of the same species. Each dot represents a different *Protium* species and different colours correspond to published taxonomic sections within the genus *Protium*.


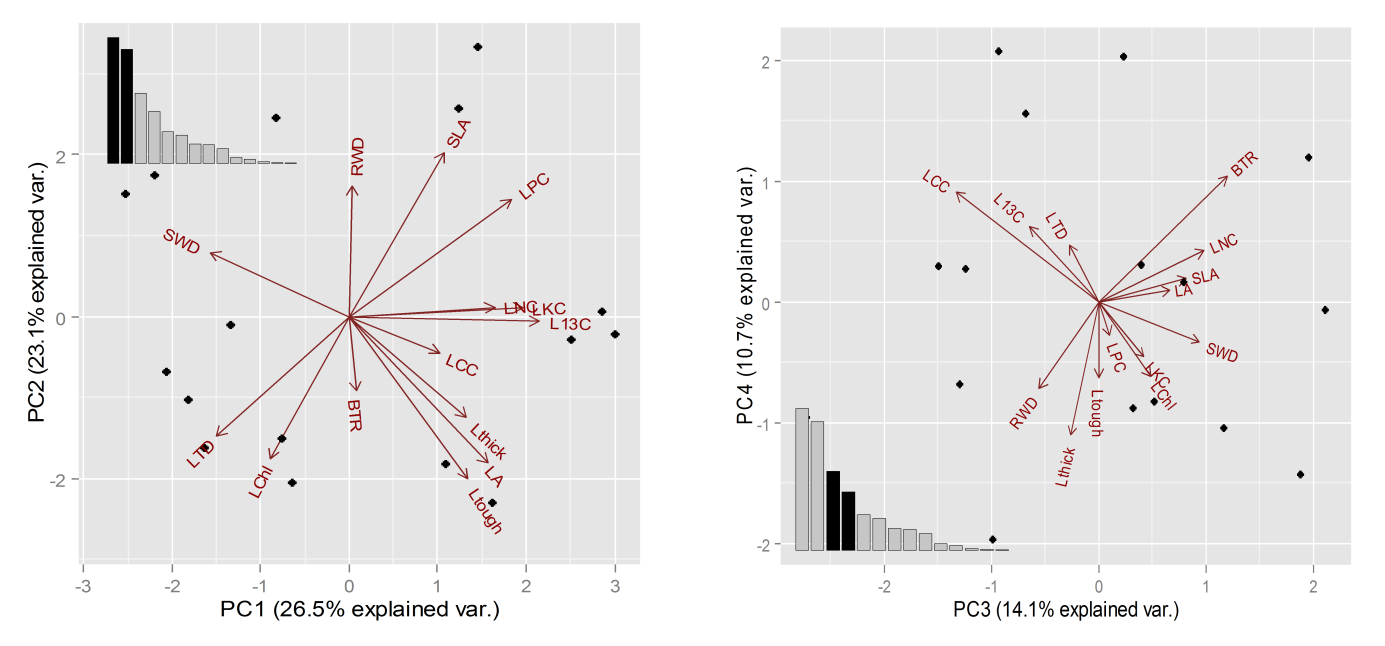
**Fig. S5**. Biplots from the principal component analyses on resource-use traits (PCA_RUT_), showing the projection of species scores on PC axes 1-2 (left figure) and axes 3-4 (right figure). Black histograms represent the eigenvalues of the projected axes. SWD/RWD: stem/root wood density; LTD: leaf tissue density; LChl: leaf chlorophyll content; BTR: bark thickness residuals; LA: leaf area; SLA: specific leaf area; LPC/LNC/LKC/LCC: leaf P/N/K/C content; L13C: leaf δ^13^C; Ltough: leaf toughness; Lthick: leaf thickness.
