# Supplementary material for: Divergent Secondary Metabolites and Habitat Filtering Both Contribute to Tree Species Coexistence in the Peruvian Amazon
Source: Front Plant Sci. 2018 Jun 19;9:836. doi: 10.3389/fpls.2018.00836 (PMC6018647; doi:10.3389/fpls.2018.00836)
Supplement: Supplementary file 3 [file Data_Sheet_2.DOCX]

**Appendix 1. Details on the secondary metabolites analyses**

***1) Biological*** ***system***

The genus *Protium* (Burseraceae) includes approximately 140 species of trees (of which 130 are found only in the Neotropics). The Burseraceae (Frankincense and Myrrh family) are well known for the production of terpene resins, and *Protium* is no exception. These compounds have been shown to have medicinal properties ([Rudiger et al. 2007](#_ENREF_17), [Siani et al. 2012](#_ENREF_20)) and are widely believed to have defensive function against pathogens and insect herbivores ([Langenheim 2003](#_ENREF_9)). *Protium* species also produce a wide array of non-volatile secondary compounds, including polymeric flavans, flavones, oxidized terpenes, and quinic acids, among others ([Lokvam and Fine 2012](#_ENREF_10), [Siani et al. 2012](#_ENREF_20), [Lokvam et al. 2015](#_ENREF_12)).

***2) Plant chemistry:***

***Chemical analysis approach:***

Although plant chemistry can vary from one individual to the other due to multiple environmental, ecological, and genetic factors, recent studies suggest that, for plants in natural conditions and exposed to natural levels of herbivore pressure, the vast majority of compounds that comprise the secondary chemical makeup of a species can be extremely consistent across individuals within a population ([Kursar et al. 2009](#_ENREF_8), [Endara et al. 2015](#_ENREF_2), [Richards et al. 2015](#_ENREF_16), [Volf et al. 2015](#_ENREF_26), [Salazar et al. 2016b](#_ENREF_19), Endara et al. 2018). Nevertheless, intraspecific chemical variation can represent a significant source of bias if not taken into account, especially if one aims to incorporate the absolute or relative concentration of secondary metabolites into an analysis. However, it is important to underline that, in our study, we are testing hypotheses that necessitate the comparison of plant secondary metabolites among *Protium* species and not at the individual or population level within species. Given that assessing secondary chemical composition for each of the saplings sampled across this study would have been an economically and logistically prohibitive endeavor, we implemented a combined subsampling approach analyzing at least six to nine individuals of each species. In concordance with other studies (see above) we found strong consistency in secondary chemical composition across individuals of the same species. Furthermore, quantitative intraspecific variation in plant chemistry was much smaller that interspecific variation. Additionally, to account for changes in secondary chemistry across leaf ontogeny, our combined subsampling approach used both mature and young, expanding leafs. The chemical composition of both young and mature leaves was ultimately combined. Only a small number of chemical compounds were found in only one developmental leaf stage (~0.8%). Finally, in order to keep our analysis conservative and reduce the amount of uncertainty inherently produced by subsampling schemes, we ultimately classified secondary compounds into presence/absence data. All compounds above the 10^th^ percentile in chromatogram peak area were considered “Present”. All compounds bellow the 10^th^ percentile in peak area where considered “absent”. This very conservative characterization was used to remove most of the uncertainty due to instrument detection limits and extraction efficiency across instruments and samples.

***Chemical analysis***

Young and mature leaf samples were collected from the same marked individuals within the permanent transects and placed immediately in enough silica gel to dry the leaf samples within a few hours. Fully dried samples where protected from high temperatures (above 30 C) and transported to the University of California, Berkeley for analysis. In order to characterize the maximum number of secondary compounds we performed separate analyses for high- and low- molecular weight metabolites. All extraction and chromatographic methods were developed *de novo* using as a starting point the work of coauthors Salazar ([Salazar et al. 2016b](#_ENREF_19), [a](#_ENREF_18)) and Lokvam ([Lokvam and Kursar 2005](#_ENREF_11), [Lokvam and Fine 2012](#_ENREF_10), [Lokvam et al. 2015](#_ENREF_12)).

***Low-molecular-weights metabolite chemistry***

***Sample extraction:***

For low-molecular weight metabolites, 100mg of dry leaf material was pulverized using a blade mill and passed through a 0.2mm sieve to standardize particle size. 75 mg of the sample (± 1 mg to the nearest 0.1 mg) was placed in a 0.22-micron spin filter (Corning Costar Spin-X, USA). Chemical compounds were extracted using centrifugal force and 150 microliters of a 1:4 solution of ethanol: dichloromethane with 0.075 mg/l of Piperine as an internal standard. Spin filters were centrifuged at 14xG for 4 minutes. Finally, flow-through was transvased to VOC autosampler vials.

***Gas Chromatography Mass Spectroscopy:***

For the analysis we used an Agilent 7890/5975C system (Agilent, USA). We injected 2.5 microliters of plant extract into a 4.0 mm ID Low Pressure Drop Precision RESTEK Inlet liner (with glass wool; Restek, USA). The inlet was kept at a constant temperature of 275 C˚. We used split injection with a 60:40 ratio. The oven used a 30m, 0.25 ID, 0.25um, HP-5ms Column (Agilent) and was programmed as follows: 85 C˚, hold for 2 min; ramp 1: 10 C˚ /min; 155 C˚, hold for 1 min; ramp 2: 6 C˚ /min; 260 C˚, hold for 1 min; ramp 3: 2 C˚ /min; 300 C˚ hold for 14 min (total run time 60 min, He as carrier). No column guard was used. MS conditions were as follows: EI source with positive ionization, 70eV, scanning range 40-550 amu, rate = 1 scan/ms. To assess carryover and retention time shifts we injected a sample of solvent containing two internal standards (Piperine and Limonine) between every *Protium* sample*.* These *“*Blank” samples were analyzed under identical chromatographic conditions described above. No significant carryover was detected.

***Data pre-processing:***

GCMS chromatograms were processed using the approach by Salazar ([Salazar et al. 2016b](#_ENREF_19), [a](#_ENREF_18)). Briefly, detection limits where standardized across samples using the internal standards. Additionally, all small peaks (peak area smaller that 2 standard deviations from the global mean peak area [average across all peaks from all samples]) where removed from the analysis. We assessed chemical similarity between all sampled species by building a mass spectra library containing all chromatographic features for each species (one library per species). The libraries of each species were then cross-referenced across all species using AMDIS (Automated Mass Spectral Deconvolution and Identification System) to identify common as well as unique features based on mass spectra, molecular weight, and expected retention time ([Davies 1998](#_ENREF_1), [Stein 1999](#_ENREF_23), [Stein et al. 2005](#_ENREF_22), [Horai et al. 2010](#_ENREF_4)). It is important to underline that this particular approach can assess chemical similarity among species independently of chemical compound identification. This methodology yielded a species-pair matrix of chemical similarity between all sampled *Protium* species.

Using the chemical similarity data from the above methodology, we performed a hierarchical clustering analysis (Ward’s algorithim, R package pvclust ([Suzuki and Shimodaira 2006](#_ENREF_24), [R Development Core Team 2015](#_ENREF_14))) to construct a dendrogram based on species chemical similarity. Subsequently, we extracted a species-pair matrix of chemical distances from the dendrogram.

Finally, the mass spectra of the different compounds in the samples were compared with NIST/EPA/NIH Databases, as well as with the primary literature. Metabolites that did not have a match from the available mass spectra databases or in the available literature were classified as unknown.

***High-molecular-weights metabolite chemistry***

***Sample Extraction:***

Leaf samples from marked trees of each *Protium* study species were further dried under moderate vacuum (100 mTorr) at ambient temperatures for 36 h. Dry sample masses were typically 200-500 mg. The entire mass of each sample was pulverized in grinding tubes using a Mini Bead Beater (BioSpec Products, Bartlesville, USA) and 3 mm steel bearings. 100 mg portions (± 1 mg to the nearest 0.1 mg) of each sample were weighed into tared 2 mL centrifuge tubes that were then fitted with a micro-stirbar. For each study species, samples of three expansion-phase and three fully expanded leaves were independently extracted 3× with hexane, 4× with 4:1 (v/v) ethanol/aq 0.5% acetic acid and 3× with 7:3 (v/v) acetone/aq 0.5% acetic acid. In each case, 1.5 mL of solvent was added to the extraction tubes and samples were stirred briskly for 10 min, then centrifuged for 2 min at 17 g. The supernatant was removed by syringe. For each sample, aqueous ethanol and acetone extracts were combined in tared vials and dried first under a stream of nitrogen at 37˚C and then under moderate vacuum for 36 h. The mass of each extract was recorded before analysis by high performance liquid chromatography (HPLC).

***High Performance Liquid Chromatography:***

HPLC analyses were carried out using two systems. The first was a Hitachi LaChrom Elite (Hitachi High Technologies America, Pleasanton, USA) equipped with a photodiode array (PDA) detector (L-2455) configured in tandem with an evaporative light-scattering (ELS) detector (SEDEX 75, S.E.D.E.R.E., Alfortville, France). The second was an Agilent 1100 system configured for electro-spray ionization mass spectrometry (ESIMS) using an ion trap mass detector (LCQ Fleet, Thermo Scientific, Waltham, MA, USA). For each analyte, PDA provided absorption data, ELS provided relative abundance data and ESIMS provided molecular weight data. In all cases, separation was done using an Atlantis T3 2 × 150 mm 3µ ODS HPLC column (Waters Corp., Milford, MA, USA) that was maintained at 40˚C.

HPLC samples were prepared by dissolving the extracts in a standard solution at the rate of 100 µg/µL. The standard solution was a 1:1 mixture of methanol and dimethyl sulfoxide that was acidified with 0.1% (v/v) formic acid and that contained the following (in order of elution) retention time standards (all 200 mM concentration): gallic acid, 4-hydroxy benzoic acid, sinapinic acid, flavone, chrysin and α-tocopherol. Injection volumes were 2 (PDA/ELS analyses) or 3 (ESIMS analyses) µL.

All HPLC analyses were carried out with a solvent system consisting of (**A**) a 1:1 mixture of methanol and acetonitrile and (**B**) water, both acidified with 0.1% (v/v) formic acid. A conservative elution program was employed. It had the following linear gradient steps. At time (t, min) = 0, 5% **A** in **B**; at t = 10, 15% **A** in **B**; at t = 60, 30% **A** in **B**; at t = 75, 70% **A** in **B**; at t = 100, 95% **A** in **B**; at t = 135, 100% **A**. This was followed by an isocratic step of 100% **A** to t = 150. With this gradient elution program, all phenolic compounds eluted by 80 min and saponins eluted by 110 min. PDA data were collected between 250 and 700 nm; ESIMS data were collected in the negative ion mode from t = 0-80, positive ion mode t = 80-114 and negative ion mode t = 114-150.

***Data pre-processing:***

HPLC raw data was processed using MZmine ([Pluskal et al. 2010](#_ENREF_13)). Each chromatogram was subject to the following workflow: (a) remove baseline noise (baseline correction *via* Savitzky-Golay filter, ([Katajamaa and Orešič 2007](#_ENREF_6))), (b) mass list construction (mass detection via Recursive Threshold Mass detector, ([Katajamaa and Orešič 2005](#_ENREF_5))), (c) construction of single chromatograms (Chromatogram Builder), (e) single chromatogram deconvolution (chromatogram deconvolution via Wavelets –XCMS-, ([Smith et al. 2006](#_ENREF_21))), (f) signal intensity normalization (Standard Compounds Normalizer), (g) peak alignment (RANSAC aligner), (h) secondary search of common compounds (Gap Filling/peak finder), (i) adduct detection (Adduct Search), (j) polymeric compound detection (CAMERA tool, ([Kuhl et al. 2011](#_ENREF_7))), (k) peak list clean-up (Duplicate filter), and (l) manual check of peak list. Finally, mirroring the approach from the low molecular weight metabolite analysis, all small peaks (peak area smaller that 2 standard deviations from the global mean peak area [average across all peaks from all samples]) where removed from the analysis. The final peak list was used to construct a plant-by-metabolite matrix.

**References**

Davies, T. 1998. The new automated mass spectrometry deconvolution and identification system (AMDIS). Spectroscopy Europe 10:24-27.

Endara, M. J., A. Weinhold, J. E. Cox, N. L. Wiggins, P. D. Coley, and T. A. Kursar. 2015. Divergent evolution in antiherbivore defences within species complexes at a single Amazonian site. Journal of Ecology 103:1107-1118.

Endara, M.-J., Coley, P.D., Wiggins, N.L., Forrister, D.L., Younkin, G.C., Nicholls, J.A., Pennington, R.T., Dexter, K.G., Kidner, C.A., Stone, G.N. and Kursar, T.A. (2018). Chemocoding as an identification tool where morphological- and DNA-based methods fall short: Inga as a case study. New Phytol. doi:10.1111/nph.15020

Fine, P., D. C. Daly, and K. M. Cameron. 2005. The contribution of edaphic heterogeneiyt to the evolution and diversity of burseracear trees in the western Amazon. Evolution 59:1464-1478.

Horai, H., M. Arita, S. Kanaya, Y. Nihei, T. Ikeda, K. Suwa, Y. Ojima, K. Tanaka, S. Tanaka, and K. Aoshima. 2010. MassBank: a public repository for sharing mass spectral data for life sciences. Journal of mass spectrometry 45:703-714.

Katajamaa, M. and M. Orešič. 2005. Processing methods for differential analysis of LC/MS profile data. BMC bioinformatics 6:1.

Katajamaa, M. and M. Orešič. 2007. Data processing for mass spectrometry-based metabolomics. Journal of chromatography A 1158:318-328.

Kuhl, C., R. Tautenhahn, C. Bottcher, T. R. Larson, and S. Neumann. 2011. CAMERA: an integrated strategy for compound spectra extraction and annotation of liquid chromatography/mass spectrometry data sets. Analytical chemistry 84:283-289.

Kursar, T. A., K. G. Dexter, J. Lokvam, R. T. Pennington, J. E. Richardson, M. G. Weber, E. T. Murakami, C. Drake, R. McGregor, and P. D. Coley. 2009. The evolution of antiherbivore defenses and their contribution to species coexistence in the tropical tree genus Inga. Proceedings of the National Academy of Sciences of the United States of America 106:18073-18078.

Langenheim, J. H. 2003. Plant resins: chemistry, evolution, ecology, and ethnobotany. Timber Press Portland, OR.

Lokvam, J. and P. V. A. Fine. 2012. An oxidized squalene derivative from Protium subserratum Engl.(Engl.) growing in Peru. Molecules 17:7451-7457.

Lokvam, J. and T. A. Kursar. 2005. Divergence in structure and activity of phenolic defenses in young leaves of two co-occurring Inga species. Journal of Chemical Ecology 31:2563-2580.

Lokvam, J., M. R. Metz, G. R. Takeoka, L. Nguyen, and P. V. A. Fine. 2015. Habitat-specific divergence of procyanidins in Protium subserratum (Burseraceae). Chemoecology 25:293-302.

Pluskal, T., S. Castillo, A. Villar-Briones, and M. Orešič. 2010. MZmine 2: modular framework for processing, visualizing, and analyzing mass spectrometry-based molecular profile data. BMC bioinformatics 11:1.

R Development Core Team. 2015. R: A language and environment for statistical computing. R Foundation for Statistical Computing, Vienna, Austria. ISBN 3-900051-07-0, URL <http://www.R-project.org>.

Rankin-de-Morona, J. M., G. T. Prance, R. W. Hutching, M. F. Silvia, W. A. Rodrigues, and M. E. Uehling. 1992. Preliminary results of a large-scale tree inventory of upland rain forest in the central Amazon. Acta Amaz. 22:493-534.

Richards, L. A., L. A. Dyer, M. L. Forister, A. M. Smilanich, C. D. Dodson, M. D. Leonard, and C. S. Jeffrey. 2015. Phytochemical diversity drives plant-insect community diversity. Proceedings of the National Academy of Sciences of the United States of America 112:10973-10978.

Rudiger, A. L., A. C. Siani, and V. F. Veiga-Junior. 2007. The chemistry and pharmacology of the South America genus Protium Burm, f.(Burseraceae). Pharmacognosy Reviews 1:93-104.

Salazar, D., A. Jaramillo, and R. J. Marquis. 2016a. Chemical similarity and local community assembly in the species rich tropical genus Piper. Ecology 97:3176-3183.

Salazar, D., A. Jaramillo, and R. J. Marquis. 2016b. The impact of plant chemical diversity on plant–herbivore interactions at the community level. Oecologia 181:1199-1208.

Siani, A. C., M. J. Nakamura, M. R. R. Tappin, S. S. Monteiro, A. C. Guimarães, and M. F. S. Ramos. 2012. Chemical composition of South American burseraceae non-volatile oleoresins and preliminary solubility assessment of their commercial blend. Phytochemical Analysis 23:529-539.

Smith, C. A., E. J. Want, G. O'Maille, R. Abagyan, and G. Siuzdak. 2006. XCMS: processing mass spectrometry data for metabolite profiling using nonlinear peak alignment, matching, and identification. Analytical chemistry 78:779-787.

Stein, S., D. Mirokhin, D. Tchekhovskoi, and G. Mallard. 2005. The NIST mass spectral search program for the NIST/EPA/ NIH mass spectral library. Scientific Instrument Services, New Jersey, USA.

Stein, S. E. 1999. An integrated method for spectrum extraction and compound identification from gas chromatography/mass spectrometry data. Journal of the American Society for Mass Spectrometry 10:770-781.

Suzuki, R. and H. Shimodaira. 2006. Pvclust: an R package for assessing the uncertainty in hierarchical clustering. Bioinformatics 22:1540-1542.

ter Steege, H. and N. C. A. Pitman and D. Sabatier and C. Baraloto and R. P. Salomão and J. E. Guevara and O. L. Phillips and C. V. Castilho and W. E. Magnusson and J. F. Molino and A. Monteagudo and P. N. Vargas and J. C. Montero and T. R. Feldpausch and E. N. H. Coronado and T. J. Killeen and B. Mostacedo and R. Vasquez and R. L. Assis and J. Terborgh and F. Wittmann and A. Andrade and W. F. Laurance and S. G. W. Laurance and B. S. Marimon and B. H. Marimon Jr and I. C. G. Vieira and I. L. Amaral and R. Brienen and H. Castellanos and D. C. López and J. F. Duivenvoorden and H. F. Mogollón and F. D. D. A. Matos and N. Dávila and R. García-Villacorta and P. R. S. Diaz and F. Costa and T. Emilio and C. Levis and J. Schietti and P. Souza and A. Alonso and F. Dallmeier and A. J. D. Montoya and M. T. F. Piedade and A. Araujo-Murakami and L. Arroyo and R. Gribel and P. V. A. Fine and C. A. Peres and M. Toledo and G. A. Aymard C and T. R. Baker and C. Cerón and J. Engel and T. W. Henkel and P. Maas and P. Petronelli and J. Stropp and C. E. Zartman and D. Daly and D. Neill and M. Silveira and M. R. Paredes and J. Chave and D. D. A. Lima Filho and P. M. Jørgensen and A. Fuentes and J. Schöngart and F. C. Valverde and A. Di Fiore and E. M. Jimenez and M. C. P. Mora and J. F. Phillips and G. Rivas and T. R. Van Andel and P. Von Hildebrand and B. Hoffman and E. L. Zent and Y. Malhi and A. Prieto and A. Rudas and A. R. Ruschell and N. Silva and V. Vos and S. Zent and A. A. Oliveira and A. C. Schutz and T. Gonzales and M. T. Nascimento and H. Ramirez-Angulo and R. Sierra and M. Tirado and M. N. U. Medina and G. Van Der Heijden and C. I. A. Vela and E. V. Torre and C. Vriesendorp and O. Wang and K. R. Young and C. Baider and H. Balslev and C. Ferreira and I. Mesones and A. Torres-Lezama and L. E. U. Giraldo and R. Zagt and M. N. Alexiades and L. Hernandez and I. Huamantupa-Chuquimaco and W. Milliken and W. P. Cuenca and D. Pauletto and E. V. Sandoval and L. V. Gamarra and K. G. Dexter and K. Feeley and G. Lopez-Gonzalez and M. R. Silman. 2013. Hyperdominance in the Amazonian tree flora. Science 342.

Volf, M., J. Hrcek, R. Julkunen-Tiitto, and V. Novotny. 2015. To each its own: Differential response of specialist and generalist herbivores to plant defence in willows. Journal of Animal Ecology 84:1123-1132.
