# Supplementary material for: Divergent Secondary Metabolites and Habitat Filtering Both Contribute to Tree Species Coexistence in the Peruvian Amazon
Source: Front Plant Sci. 2018 Jun 19;9:836. doi: 10.3389/fpls.2018.00836 (PMC6018647; doi:10.3389/fpls.2018.00836)
Supplement: Supplementary file 8 [file Data_Sheet_7.DOCX]

**Appendix S6**. R code used for statistical analyses

library(vegan) ; library(usdm) ; library(BiodiversityR) ; library(ggbiplot)

library(ape) ; library(ade4) ; library(spacodiR) ; library(car)

## Functional traits data

#************************

## Secondary metabolites (SM) and abundance of Protium species with SM data available:

SM <- as.data.frame(read.table("AppendixS2.xls",h=T,row.names=1))

SM <- SM[,-c(1:5)]

SM <- as.data.frame(t(SM))

dim(SM)

# Protium species presence-absence data

Protium <- as.data.frame(read.table("AppendixS1.txt",h=TRUE,row.names=1))

Protium.SM <- Protium[,colnames(Protium)%in%rownames(SM)]

Protium.SM <- Protium.SM[specnumber(Protium.SM)>=2,]

Protium.SM <- Protium.SM[,colSums (Protium.SM)>0 ]

Protium.SM <- Protium.SM[specnumber(Protium.SM)>=2,]

dim(Protium.SM)

colnames(Protium.SM)

## Scaling values in SM from 0 to 1:

for(i in 1:ncol(SM)){ SM[,i] = as.numeric((SM[,i]/max(SM[,i]))) }

## Resource use traits (RUT) and abundance of Protium species with RUT data available:

# RUT data

RUT <- as.data.frame(read.table("AppendixS3.xls",h=T,row.names=1))

# standardization + normalization of RUT:

for(i in 1:ncol(RUT)){ RUT[,i] <- as.numeric((RUT[,i]-mean(RUT[,i]))/sd(RUT[,i])) }

dim(FT)

par(mfrow=c(3,5), mex=0.3)

for(i in 1:ncol(RUT)){hist(RUT[,i],col="blue")}

par(mfrow=c(1,1))

# PCA on RUT data and RUT variables significantly associate to each PC axis (Table S2 in supplementary material)

pca <- rda(RUT)

barplot(pca$CA$eig)

library(BiodiversityR)

(sign <- PCAsignificance(pca)) ## axes explaining more variation than expected by a broken stick model

TableS2 <- as.data.frame(matrix(0,nrow=ncol(RUT),ncol=4))

rownames(TableS2) <- colnames(RUT)

colnames(TableS2) <- c("PCaxis1","PCaxis2","PCaxis3","PCaxis4")

for(j in 1:4){

for(i in 1:nrow(Table1)){

TableS2[i,j] <- paste(round(cor(RUT[,i],pca$CA$u[,j]),3),round(cor.test(RUT[,i],pca$CA$u[,j])$p.value,3))

}

}

TableS2

str(pca)

## biPCA on RUT using the ggbiplot function:

prc <- prcomp(RUT, center=T, scale=T, retx=T)

#groups = c(rep("L",12),rep("I",9),rep("M",9),rep("T",6))

summary(prc)

varimax3 <- varimax(prc$rotation[,1:3])

print(prc)

plot(prc, type = "l")

predict(prc, newdata=tail(RUT, 2))

library(devtools)

#install_github("ggbiplot", "vqv")

library(ggbiplot)

g <- ggbiplot(prc, choices = c(1,2), obs.scale = 1, var.scale = 1,

ellipse = TRUE, circle = TRUE)

g <- g + scale_color_discrete(name = '')

g <- g + theme(legend.direction = 'horizontal',

legend.position = 'top')

print(g)

## Protium species abundance data:

Protium.RUT <- Protium [,colnames(Protium)%in%rownames(RUT)]

Protium.RUT <- Protium.RUT [rownames(Protium.RUT)%in%rownames(Protium.SM),]

Protium.RUT <- Protium.RUT[specnumber(Protium.RUT)>=2,]

Protium.RUT <- Protium.RUT[,colSums (Protium.RUT)>0 ]

Protium.RUT <- Protium.RUT[specnumber(Protium.RUT)>=2,]

dim(Protium.RUT)

Protium.SM <- Protium.SM [rownames(Protium.SM )%in%rownames(Protium.RUT),]

Protium.SM <- Protium.SM [specnumber(Protium.SM )>=2,]

Protium.SM <- Protium.SM [,colSums (Protium.SM )>0 ]

Protium.SM <- Protium.SM [specnumber(Protium.SM )>=2,]

dim(Protium.SM)

rownames(Protium.SM)%in%rownames(Protium.RUT)

## Soil data:

##************

SOIL <- as.data.frame(read.table("AppendixS4.xls",h=TRUE,row.names=1))

soil <- SOIL[rownames(SOIL)%in%rownames(Protium.RUT),]

soil <- soil[,3:ncol(soil)]

head(soil);dim(soil)

## standardize + normalize soil data:

for(i in 1:ncol(soil)){ soil[,i]=decostand(soil[,i],"standardize") }

for(i in 1:ncol(soil)){

e = powerTransform (soil[,i]+sqrt(min(soil[,i], na.rm=T)^2)+0.00000001)

soil[,i] = bcPower (soil[,i]+sqrt(min(soil[,i], na.rm=T)^2)+0.00000001,e$lambda)

}

## VIF calculation of each normalized variable

## Rem: Not to be calculate when performing the PCA!

vifstep=vifstep(soil,th=10) ; vifstep

## Removing each variable with VIF <= 10:

soil=soil[,-which(colnames(soil)%in%vifstep@excluded)]

head(soil) ; dim(soil)

soil=as.matrix(soil);colnames(soil) <- colnames(soil)

head(soil);dim(soil)

par(mfrow=c(4,4), mex=0.3) ; for(i in 1:ncol(soil)){hist(soil[,i], col="green3", main=colnames(soil)[i])}

par(mfrow=c(1,1))

### Spatial coordinates

##*********************

coord = SOIL[,1:2]

coord=coord[rownames(coord)%in%rownames(Protium.RUT),]

## removing one distant plots for SM:

plot(coord)

coord <- coord[-nrow(coord),]

plot(coord)

### Re-adjusting matrices to ensure that lines correspond to the same plots

Protium.RUT <- Protium.RUT[rownames(Protium.RUT)%in%rownames(coord),]

Protium.SM <- Protium.SM[rownames(Protium.SM)%in%rownames(coord),]

dim(Protium.RUT);dim(Protium.SM)

rownames(Protium.RUT);rownames(Protium.SM)

coord <- coord[rownames(coord)%in%rownames(Protium.RUT),]

soil <- soil[rownames(soil)%in%rownames(Protium.RUT),]

habs <- as.numeric(SOIL[rownames(SOIL)%in%rownames(Protium.RUT),][,3])

SM <- SM[rownames(SM)%in%colnames(Protium.SM),]

dim(Protium.RUT);dim(Protium.SM);dim(SM);dim(RUT);dim(soil);dim(coord)

order.t <- c()

for(i in 1:nrow(RUT)){

order.t <- c(order.t, which(rownames(RUT)%in%colnames(Protium.RUT)[i]))

}

order.t

RUT <- RUT[order.t,]

order.t <- c()

for(i in 1:nrow(SM)){

order.t <- c(order.t, which(rownames(SM)%in%colnames(Protium.SM)[i]))

}

order.t

SM <- SM[order.t,]

## PCA on soil data (read next section to reach 19 plots only):

pca=rda(soil)

PCAsignificance(pca)

## PCA using the ggbiplot function:

prc <- prcomp( soil, center=T, scale=T, retx=T)

groups = habs

groups[which(groups==3)] <- "WS"

groups[which(groups==2)] <- "TF"

summary(prc)

varimax3 <- varimax(prc$rotation[,1:3])

print(prc)

plot(prc, type = "l")

predict(prc, newdata=tail(soil, 2))

library(devtools)

#install_github("ggbiplot", "vqv")

library(ggbiplot)

g <- ggbiplot(prc, choices=c(1,2), obs.scale = 1, gvar.scale = 1, ellipse = TRUE, circle = TRUE)

g <- g + scale_color_discrete(name = '')

g <- g + theme(legend.direction = 'horizontal',

legend.position = 'top')

print(g)

### Testing within-plot SM and RUT dissimilarity

#***********************************************

# Species.Ab = species abundance matrix (Protium)

# Traits = Traits data matrix

# coph.phylo = phylogenetic tree (in "nexus" or "newick" format)

# nr = number of simulations of null dissimilarity values

# distance = distance metric used

# model = null model used to generate null dissimilarity values

Species.Ab = Protium.SM

# or:

Species.Ab = Protium.RUT

colnames(Species.Ab)

rownames(Traits)

Alpha <- function(Species.Ab, Traits, habs, coph.phylo, model, nr, distance, CD, FD)

{

Species.Ab[Species.Ab>0] <- 1

vec.species <- c()

SAD <- colSums(Species.Ab)[order(colSums(Species.Ab))]

for(z in 1:length(SAD)){ vec.species <- c(vec.species,rep(names(SAD)[z],as.numeric(SAD[z]))) }

# obs. dissim:

for(i in 1:nrow(Species.Ab)){

species.ab.i <- Species.Ab[i,]

sp <- which(Species.Ab[i,]>0)

if(is.vector(Traits) == "TRUE") { O <- Traits[sp] }

if(is.vector(Traits) == "FALSE"){ O <- Traits[sp,]}

D.obs <- as.matrix(vegdist(O,method=distance))

D.obs <- D.obs[lower.tri(D.obs)=="TRUE"]

TEST.plot[i,1] <- mean(D.obs)

# expected dissim:

if(CD == 1){ vec.chemicals <- colnames(Traits) }

for(k in 2:nr){

u=i ; species.ab.u <- species.ab.i

samp <- c() ; a1=0

while(a1==0){

samp <- c(samp,sample(sample(vec.species))[1])

if(length(samp)>=length(sp)&length(table(samp))==length(sp)){a1=1}

}

sp2 <- which(colnames(Species.Ab)%in%samp)

#samp <- sample(sample(colnames(species.ab.h)),specnumber(Species.Ab[u,]),replace=FALSE)

samp <- sample(sample(colnames(Species.Ab)),specnumber(Species.Ab[u,]),replace=FALSE)

sp2 <- which(colnames(Species.Ab)%in%samp)

# SM dissim

if(CD == 1){

B <- as.data.frame(matrix(0,nrow=length(samp),ncol=ncol(Traits)))

for(cd in 1:length(samp)){B[cd,] <- Traits[which(rownames(Traits)%in%samp[cd]),]}

D.exp <- as.matrix(vegdist(as.matrix(B),method=distance)) ; D.exp <- D.exp[lower.tri(D.exp)=="TRUE"] ; EXP <- mean(D.exp)

}

# RUT dissim.

if(is.vector(Traits) == "TRUE") { T <- Traits[sp2] } ; if(is.vector(Traits) == "FALSE"){ T <- Traits[sp2,]}

D.exp <- as.matrix(vegdist(as.matrix(T),method=distance)) ; D.exp <- D.exp[lower.tri(D.exp)=="TRUE"] ; EXP <- mean(D.exp)

TEST.plot[u,k] <- EXP

}

}

# Store p-values

p.values1 = c() ; p.values2 = c()

for(i in 1:nrow(Species.Ab)){

p.values1 = c(p.values1, length(TEST.plot[i,2:nr][TEST.plot[i,2:nr]>TEST.plot[i,1]])/nr )

p.values2 = c(p.values2, length(TEST.plot[i,2:nr][TEST.plot[i,2:nr]<TEST.plot[i,1]])/nr )

}

# vector of size N (number of plots) of standard deviation of the nr O-E values

# among simulations (for each plot):

SD.plots <- c()

for(q in 1:nrow(TEST.plot)){SD.plots <- c(SD.plots,sd(as.numeric(TEST.plot[q,1]-as.numeric(TEST.plot[q,2:nr]))))}

# P-value of testing whether the mean [O-mean(E calculated over all simulations)]

# at the plot level is higher or lower than zero:

P2 <- length(mean.OE.overallplots.vec[mean.OE.overallplots.vec>0])/nr

## RESULTS table:

# - - - - - - - -

RESULTS<-cbind(TEST.plot[,1],as.numeric(rowMeans(TEST.plot[,2:nr],na.rm = TRUE)), TEST.plot[,1]-as.numeric(rowMeans(TEST.plot[,2:nr],na.rm = TRUE)),

SD.plots, p.values1, p.values2) ; colnames(RESULTS) <- c("OBS","mean_EXP","OBS-EXP","SD(OBS-EXP)","P-value_>","P-value_<")

list(RESULTS = RESULTS, P2)

}

## Perform the Alpha function on the SM or RUT data:

Traits <- SM

Traits <- RUT

## if analysing SM:

Traits[Traits>0] <- 1

## if analysing RUT:

#variables correlated to PCAxis 1:

ax1 <- c(2,6,9,11:14) ; colnames(Traits)[ax1]

Traits <- Traits[,ax1]

#variables correlated to PCAxis 2:

ax2 <- c(3,4,6:9,12) ; colnames(Traits)[ax2]

Traits <- Traits[,ax2]

#variables correlated to PCAxis 3:

ax3 <- c(1,8,10,11) ; colnames(Traits)[ax3]

Traits <- Traits[,ax3]

#variables correlated to PCAxis 4:

ax4 <- c(5,10) ; colnames(Traits)[ax4]

Traits <- Traits[,ax4]

nr = 1000

Species.Ab = Protium.SM;Species.Ab = Protium.RUT

CD = 1 ; FD = 0 ; distance = "jaccard" # if analyzing SM

FD = 1 ; CD = 0 ; distance = "euclidean" # if analyzing RUT

alph <- Alpha(Species.Ab=Species.Ab,Traits=Traits,CD=CD,FD=FD,nr=nr,distance=distance)

## Turn-over analyses

#********************

## Function "Beta" to test functional turn-over:

# space.detrend => if = 1 => trait/phylogenetic turn-over is calculated on the

# residuals of the turn-over regressed on log(spatial distance)

# dist.trait = distance metric used

Beta <- function(Species.Ab, Traits, coord, soil.matrix, dist.trait, space.detrend)

{

T <- Traits

if(dist.trait == "jaccard"){T = vegdist(T,method="jaccard")}

S <- spacodi.calc(sp.plot=t(Species.Ab), sp.traits = T, pairwise=TRUE)

TAUst <- as.dist(S2$pairwise.TAUst)

# Spatial distance matrix:

geodist <- as.matrix(dist(coord)) ; geodist <- log(as.dist(geodist)+1)

# Edaphic distance matrix:

soildist <- as.dist(dist(soil.matrix,method="euclidean"))

# Store all distance data in a single matrix:

M <- cbind(TAUst,geodist,soildist)

## Mantel test between all pairwise metrics

Mat.list<-list(as.dist(S$pairwise.TAUst), as.dist(geodist),as.dist(soildist) )

Mantel<-as.data.frame(matrix(0,ncol=ncol(M),nrow=ncol(M)))

for(a in 1:nrow(Mantel)){

for(b in 1:nrow(Mantel)){

if(space.detrend == 1){

if(b==3){Mantel[a,b]<-paste(round(cor(resid(lm(M[,a]~M[,4])),M[,b]),2),"(p* =",mantel.partial((Mat.list[[a]]),(Mat.list[[b]]),(Mat.list[[4]]), method = "pearson", permutations = 999)$sign,")")}

if(b!=3){Mantel[a,b]<-paste(round(cor(M[,a],M[,b]),2),"(p =",mantel.rtest((Mat.list[[a]]),(Mat.list[[b]]))$pvalue,")")}

}

if(space.detrend == 0){Mantel[a,b] <- paste(round(cor(M[,a],M[,b]),2),"(p =",mantel.rtest((Mat.list[[a]]),(Mat.list[[b]]))$pvalue,")")}

}

}

# store results

list(M = M , Mantel = Mantel,geodist = geodist, soildist = soildist)

}

Species.Ab <- Protium.SM ; Traits <- SM

#or:

Species.Ab <- Protium.FT ; Traits <- FT

# if using SM:

Traits2 <- Traits

Traits2[Traits2>0] <- 1

# if using RUT:

#variables correlated to PCAxis 1:

ax1 <- c(2,6,9,11:14) ; colnames(Traits)[ax1]

Traits2 <- Traits[,ax1]

#variables correlated to PCAxis 2:

ax2 <- c(3,4,6:9,12) ; colnames(Traits)[ax2]

Traits2 <- Traits[,ax2]

#variables correlated to PCAxis 3:

ax3 <- c(1,8,10,11) ; colnames(Traits)[ax3]

Traits2 <- Traits[,ax3]

#variables correlated to PCAxis 4:

ax4 <- c(5,10) ; colnames(Traits)[ax4]

Traits2 <- Traits[,ax4]

# soil variables associated to axes 1, 2 or 3 of the PCAsoil:

colnames(soil)

soil2 <- soil[,c(1,3,4,7,8,9,11)] ; colnames(soil2) ## variables associated to axis 1

soil2 <- soil[,c(2,4,5,9)] ; colnames(soil2) ## variables associated to axis 2

soil2 <- soil[,c(1,3,7,9)] ; colnames(soil2) ## variables associated to axis 3

soil2 <- soil[,-c(1,3,7,9)] ; colnames(soil2) ## variables associated to axis 3

soil2 <- soil

# perform the Beta function to get the TAUst values for SM or RUT data, as well as the corresponding spatial soil distance values:

Beta(Species.Ab=Species.Ab,Traits=Traits2,coord=coord,soil.matrix=soil2,dist.trait=dist.trait,space.detrend=space.detrend)
